# Role of transporters in the distribution of platinum-based drugs

**DOI:** 10.3389/fphar.2015.00085

**Published:** 2015-04-24

**Authors:** Saliha Harrach, Giuliano Ciarimboli

**Affiliations:** ^1^Experimental Nephrology, Medical Clinic D, University of Münster, University Hospital MünsterMünster, Germany; ^2^Interdisciplinary Center for Clinical Research (IZKF), University of Münster, University Hospital MünsterMünster, Germany

**Keywords:** cisplatin, oxaliplatin, transporters, side effects, uptake

## Abstract

Platinum derivatives used as chemotherapeutic drugs such as cisplatin and oxaliplatin have a potent antitumor activity. However, severe side effects such as nephro-, oto-, and neurotoxicity are associated with their use. Effects and side effects of platinum-based drugs are in part caused by their transporter-mediated uptake in target and non target cells. In this mini review, the transport systems involved in cellular handling of platinum derivatives are illustrated, focusing on transporters for cisplatin. The copper transporter 1 seems to be of particular importance for cisplatin uptake in tumor cells, while the organic cation transporter (OCT) 2, due to its specific organ distribution, may play a major role in the development of undesired cisplatin side effects. In polarized cells, e.g., in renal proximal tubule cells, apically expressed transporters, such as multidrug and toxin extrusion protein 1, mediate secretion of cisplatin and in this way contribute to the control of its toxic effects. Specific inhibition of cisplatin uptake transporters such as the OCTs may be an attractive therapeutic option to reduce its toxicity, without impairing its antitumor efficacy.

In order to reach sufficiently high concentrations, intracellular active drugs must cross the plasma membrane to reach their targets. This process, especially in the case of hydrophilic drugs, is mediated by specialized plasma membrane proteins called transporters. It is well known that efflux transporters are involved in the development of drug resistance (Renes et al., 2000). Only recently, it has become evident that uptake transporters not only are able to mediate drug effects but also their side effects (Ciarimboli, 2011). In this mini-review, we will focus on transporter systems for platinum-based drugs.

Cisplatin was the first platinum-based drug that revolutionized the treatment of neoplastic diseases. For example, before the introduction of cisplatin as chemotherapeutic agent, testicular cancer was associated with a survival rate of only 5%. Today, treatment of this cancer with a combination of new surgical techniques and cisplatin chemotherapy allows to achieve a cure rate of over 90% (Einhorn, 2002). Currently, cisplatin is widely used for the therapy of solid tumors. However, its use is limited by severe side effects such as nephro- and ototoxicity and peripheral neurotoxicity (Rabik and Dolan, 2007). Therefore, there is a need to put an effort in developing less toxic platinum derivatives. The US Food and Drug Administration has approved carboplatin and oxaliplatin as chemotherapeutic agents. Nevertheless, because of its superior efficacy, cisplatin remains the first-line treatment for several solid cancers.

Treatment with cisplatin causes an acute and/or chronic nephrotoxicity in about one-third of patients even under co-treatment with diuretics and pre-hydration (Pabla and Dong, 2008). The clinical manifestations of cisplatin nephrotoxicity are a decreased glomerular filtration rate, increased serum creatinine level, reduced serum magnesium and potassium levels (Pabla and Dong, 2008), and the development of proteinuria of tubular and glomerular origin (Daugaard, 1990). There are striking interindividual differences in susceptibility to nephrotoxicity (Goren et al., 1986).

Animal studies showed that cisplatin damages mainly the proximal tubules (Dobyan et al., 1980), where the S3 segment is highly sensitive to cisplatin toxicity and undergoes extensive necrosis *in vivo* (Price et al., 2004). In contrast, carboplatin treatment rarely results in nephrotoxicity (Bregman and Williams, 1986; Yasumasu et al., 1992; Rabik and Dolan, 2007) and nephrotoxicity has never been observed with oxaliplatin, allowing its administration without hydration (Cassidy and Misset, 2002).

Tumor therapy with cisplatin causes a hearing loss, which is an unresolved clinical problem especially in pediatric patients (Skinner, 2004). By early onset, cisplatin therapy may lead to a delayed speech development with serious consequences for social and educational development (Skinner, 2004). Ototoxicity appears with an incidence between 23 and 50% in adults and greater than 50% in children (Rabik and Dolan, 2007). It manifests clinically with bilateral symmetrical high-frequency sensorineural hearing loss, ear pain or tinnitus (Reddel et al., 1982). In the cochlea cisplatin accumulates in the hair cells of the basal turn of Corti organ (where high-frequency sounds are processed), in the spiral ganglion cells, and in the lateral wall tissues (spiral ligament and stria vascularis) (Rybak and Ramkumar, 2007). Upon cisplatin accumulation, these cells undergo apoptosis (Rybak and Ramkumar, 2007). Carboplatin and oxaliplatin are much less ototoxic than cisplatin (Musial-Bright et al., 2011). Interestingly, intravenous application of oxaliplatin (16.6 mg/kg) in guinea pigs resulted in cochlear drug concentrations lower than those measured upon treatment with equimolar cisplatin (12.5 mg/kg), suggesting that lower cochlear oxaliplatin accumulation is the reason for its lower ototoxicity compared with cisplatin (Hellberg et al., 2009).

Most patients treated with cisplatin develop a dose-related large fiber sensory neuropathy, caused by cisplatin accumulation in the dorsal root ganglia (DRG) (Meijer et al., 1999; Albers et al., 2011). The symptoms of peripheral neurotoxicity may appear already 1 month after initiating treatment and include paresthesia, ataxia, numbness, reduced vibration, and joint position sensations and diminished or absent muscle stretch reflexes (Roelofs et al., 1984; Thompson et al., 1984; Cano et al., 1998; Albers et al., 2011). Cisplatin toxicity seems to affect large diameter neurons and proprioceptive sensory modalities (reviewed in Screnci and McKeage, 1999), where platinum accumulation within DRG leads to atrophy or loss of peripheral sensory neurons (Liu et al., 2009). Carboplatin is less neurotoxic than cisplatin. Neurotoxicity appears in only 4–6% of patients after administration of high-dose carboplatin therapy (Amptoulach and Tsavaris, 2011). Conversely, antitumor treatment with oxaliplatin causes significant acute and chronic peripheral sensory neuropathy (Amptoulach and Tsavaris, 2011). Acute neurotoxic effects develop in 85–95% of patients treated with oxaliplatin and may result from the impairment of voltage-gated sodium channels, reduction in intracellular calcium and of growth-associated protein-43 expression, or induction of oxidative stress (Adelsberger et al., 2000; Gamelin et al., 2002; Carozzi et al., 2010; Amptoulach and Tsavaris, 2011; Takeshita et al., 2011). The symptoms of oxaliplatin neurotoxicity include paresthesia in the extremities and the perioral region, and dysfunction of fine sensory-motor coordination (Cavaletti and Zanna, 2002; Hartmann and Lipp, 2003; Amptoulach and Tsavaris, 2011). These symptoms are exacerbated by cold exposure (Amptoulach and Tsavaris, 2011). It is evident that oxaliplatin neurotoxicity impairs quality of daily life.

The antitumor effects of platinum derivatives seem to derived from the formation of intra- and inter-strand crosslinks with DNA (Wang and Lippard, 2005).

Focusing on cisplatin, the molecular species involved in this process seems to be aquaions, which are supposed to be formed upon cytosolic cisplatin hydrolysis driven by the lower intracellular Cl^−^ concentration. The cisplatin aquaions are strongly electrophilic and bind DNA and sulfhydryl groups of proteins.

Since cisplatin is a hydrophilic drug (Houjou et al., 1996), it cannot easily cross the plasma membrane. In the kidneys cisplatin is free filtered in the glomerulus and also secreted into the urine (Jacobs et al., 1984). These observations suggested that the movement of cisplatin through the plasma membrane is mediated by transport proteins.

## Copper transporter 1 (Ctr1, Solute Carrier 31A1-SLC31A1)

Copper transporter 1 (Ctr1, Solute Carrier 31A1-SLC31A1) is a membrane protein that plays a significant role in the cellular cisplatin uptake (Holzer et al., 2004, 2006; Safaei and Howell, 2005; Larson et al., 2009). Down-regulation of Ctr1 extensively reduced cisplatin uptake in yeast and in mouse embryonic fibroblasts (Ishida et al., 2002; Lin et al., 2002). The natural substrate of Ctr1 is monovalent copper (Cu^+^) (Ohrvik and Thiele, 2014). Cu^+^ uptake by Ctr1 triggers transporter internalization. However, whether this phenomenon also happens upon cisplatin transport is debated (Sinani et al., 2007; Tsai et al., 2014). As observed for Cu^+^, cisplatin binds to Methionine-rich motifs of the extracellular domain of Ctr1 (Ohrvik and Thiele, 2014). Ctr1 carries out vital physiological function supplying the cell with copper, which is an essential cellular nutrient used in a broad range of enzymatic reactions. Because of its important biological role, Ctr1 is almost ubiquitously expressed and perhaps may not be the decisive transporter for specific cisplatin toxicities. Since several cell lines from human tumor samples express Ctr1-mRNA (Ciarimboli et al., 2010), this transporter could represent the uptake route of cisplatin in cancer cells. Indeed, high expression levels of Ctr1 have been associated with cisplatin therapeutic success (Lee et al., 2011; Liang et al., 2012) whereas Ctr1 mutations are associated with cisplatin resistance (Xu et al., 2012). Ctr1 has been also associated with the cellular transport of carboplatin and oxaliplatin. However, cells with a genetic deletion of Ctr1 show an uptake of oxaliplatin at concentrations higher than 2 μM, suggesting the presence of another low-affinity oxaliplatin transport system (Holzer et al., 2006).

## Copper transporter 2 (Ctr2, SLC31A2)

Copper transporter 2 (Ctr2, SLC31A2) is a copper transport protein with substantial structural homology to Ctr1. Ctr2 is mainly expressed in late endosomes and lysosomes (Blair et al., 2009), where it probably mediates the efflux of copper under conditions of low environmental copper concentration (van den Berghe et al., 2007). A similar function of Ctr2 was proposed for cisplatin (Blair et al., 2011). Studies in Ctr2-deficient mice suggested that Ctr2 functions as an indirect regulator of Cu^+^-uptake and intracellular flux by stabilizing the biosynthesis of cleaved Ctr1. The cleaved Ctr1 is a transporter form which lacks metal binding Methionine- and Histidine-rich motifs and of consequence has decreased Cu^+^ and also cisplatin uptake function (Ohrvik et al., 2013). Therefore, high expression of Ctr2 seems to be associated with resistance to the cytotoxic effect of cisplatin (Blair et al., 2009) and knockdown of Ctr2 was associated with an increased cisplatin accumulation and cytotoxicity (Blair et al., 2009, 2011).

## Copper-transporting ATPase 1 and 2 (ATP7A and ATP7B)

The P-type copper-transporting ATPases ATP7A and ATP7B are also involved in cellular cisplatin handling. These transporters play an important role in regulating the cellular copper levels, because too high intracellular copper concentrations are toxic for the cell. Inactivation of these transporters, as present for example in Menkes' disease (inactivation of ATP7A) and in Wilson's disease (inactivation of ATP7B), is associated with copper deficiency because of impaired copper efflux from enterocytes into the blood or massive cellular copper overload, respectively (Gupta and Lutsenko, 2009). While ATP7A is mainly expressed in intestine, choroid plexus, vascular smooth muscle and endothelial cells, as well as in cerebrovascular endothelial cells (Lutsenko et al., 2007), ATP7B is principally expressed in the liver and the brain (Lutsenko et al., 2007). Regarding the transport of cisplatin, ATP7A and ATP7B mediate its efflux from the cell or its distribution to specific sub-cellular compartments (Katano et al., 2003, 2004; Samimi et al., 2004a,b; Safaei et al., 2008, 2012). For this reason, the expression of these transporters is correlated with cisplatin cellular sensitivity and resistance (Komatsu et al., 2000; Nakagawa et al., 2008): ATP7B is stronger associated with the acquisition of resistance than Ctr1 or ATP7A (Yoshizawa et al., 2007; Mangala et al., 2009). Besides cisplatin, ATP7A and B transporters also interact with carboplatin and oxaliplatin (Samimi et al., 2004b; Martinez-Balibrea et al., 2009). Even though the effects of ATP7A and B transporters on cisplatin cellular distribution are very similar to those observed for copper, platinum drugs are not readily exported after vesicular sequestration (Samimi et al., 2004b).

Interestingly, copper transport systems are expressed and active in DRG, which are sensitive to toxicity from platinum derivatives. Here, Ctr1 is expressed in large-sized neurons and ATP7A in small DRG neurons (Ip et al., 2010), suggesting that large neurons are especially sensitive and small neurons are protected from toxic effects of platinum derivatives.

## Organic cation transporters (OCT1-3, SLC22A1-3)

**Figure 1:**
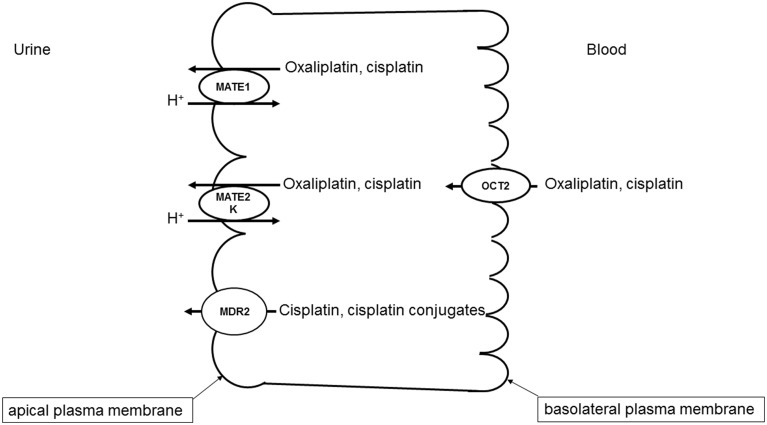
**Transport systems involved in secretion of platinum derivatives in human renal proximal tubules**. Uptake of oxaliplatin and cisplatin from the blood is mediated by hOCT2 expressed in the basolateral plasma membrane. hMATE1, hMATE2-K, and Mdr2 in the apical plasma membrane mediate the secretion of platinum derivatives into the urine.

A specific interaction of cisplatin with OCTs has also been demonstrated (Ciarimboli et al., 2005; Yonezawa et al., 2005; Filipski et al., 2008). Since OCTs have a specific organ distribution, with high renal expression, the cisplatin-OCT interaction is of special interest to explain selective organ toxicity of cisplatin. OCTs are highly expressed in excretory organs such as the liver and the kidneys, where they mediate the electrogenic uptake of their substrates in hepatocytes and proximal tubule cells, respectively (Ciarimboli, 2008). OCTs are defined as polyspecific transporters, because they can transport several unrelated substances. The driving force for the cellular transport by OCTs is the electrochemical gradient of the substrate (Ciarimboli and Schlatter, 2005). In excretory organs, OCTs mediate the first step of secretion process, consisting of substrate uptake through the basolateral plasma membrane (the blood-faced part of plasma membrane). The subsequent substrate efflux through the luminal membrane (the bile- or urine-faced part of plasma membrane) is the final secretion step, resulting in a vectorial substrate movement from the blood to the bile or urine in the liver or kidneys (Figure 1), respectively. In humans the paralogs hOCT1 and hOCT2 are specifically expressed in the basolateral membrane of hepatocytes and renal proximal tubule cells, respectively (Koepsell, 2004). Cisplatin seems to interact preferentially with hOCT2 (Ciarimboli et al., 2005), suggesting that hOCT2 is the critical transporter for renal cisplatin uptake in humans (Figure 1). Also the second- and third generation platinum-derivatives oxaliplatin (Yonezawa et al., 2006) and picoplatin (More et al., 2010), respectively, are substrates of OCTs. For the interpretation of translational studies, it is important to underline that the rodent OCT orthologs have a different organ distribution and kinetic properties compared with human OCTs: for example, in mice OCT1 is expressed in renal proximal tubules at higher level than OCT2 (Schlatter et al., 2014). Competition of OCT-mediated cisplatin transport is able to reduce cisplatin uptake (Ciarimboli et al., 2010; Wehe et al., 2014) and toxicity (Ciarimboli et al., 2005, 2010; Yonezawa et al., 2005) *in vitro* and *in vivo*. OCT2 has been demonstrated to be expressed in the mouse cochlea in hair cells of Corti organ and in the cells of the stria vascularis (Ciarimboli et al., 2010) and in mouse and human DRG (Sprowl et al., 2013), structures that are specially sensitive to toxicity by platinum-derivatives. In animal models it has been demonstrated that OCTs are critical mediators of cisplatin ototoxicity (Ciarimboli et al., 2010) and oxaliplatin peripheral neurotoxicity (Sprowl et al., 2013). Currently, the role of genetic variations of *SLC22A2* for the development of cisplatin-induced nephrotoxycity is debated: some publications describe an association of the *SLC22A2* non-synonymous single nucleotide polymorphism (SNP) 808 G/T (resulting in an exchange of alanine to serine at position 270) with a protection against cisplatin nephrotoxicity (Filipski et al., 2009; Zhang and Zhou, 2012), other studies however could not confirm this finding (Tzvetkov et al., 2011). Recent results suggest that the SNP 808 G/T confers a protection against cisplatin ototoxicity in a pediatric population (Lanvers-Kaminsky et al., 2015). Therefore, it could be assumed that inhibition of hOCT2 by a competitor could prevent cisplatin-induced side effects. Indeed, there are several indications that patient co-treatment with cimetidine protects the kidneys from cisplatin nephrotoxicity (Sleijfer et al., 1987; Zhang and Zhou, 2012). However, such a protective therapeutic option is acceptable, if the uptake of cisplatin into target tumor cells is not compromised. In animal models and in patients, it has been demonstrated that the expression of OCTs in tumor cells is down-regulated by epigenetic modifications (Schaeffeler et al., 2011; Chen et al., 2013; Yang et al., 2013). Moreover, studies in an animal model have shown that co-treatment with cimetidine did not change the antitumor efficacy of cisplatin (Katsuda et al., 2010). For these reasons, inhibition of OCTs remains an attractive option to protect patients from cisplatin side effects.

Comparing the transport properties of OCTs and Ctr1, it was underlined that Ctr1 is an equilibrative transporter. On the other side, although OCTs are not active transporters in the sense of their activity being directly dependent on ATP (primary active) or a trans-membrane ion flux (secondary active), the plasma membrane potential creates both an electrical and a concentration gradient for their cationic substrates. This allows an accumulation of platinum derivatives in the cells to intracellular concentrations exceeding the extracellular level (Yonezawa and Inui, 2011). However, the affinities of OCT2 and Ctr1 for cisplatin seem to be similar [11 (Filipski et al., 2008) and 17 μM (Liang et al., 2009), respectively].

While OCTs mediate the basolateral uptake of cisplatin in renal proximal tubule cells, other transporters expressed in the apical cell membrane are involved in the cisplatin secretion into the urine (Figure 1).

## Multidrug and toxin extrusion protein 1 (MATE1, SLC47A1)

Several evidences indicate that MATE1 mediates secretion of cisplatin into the urine. Mice with genetic deletion of MATE1 are more sensitive to cisplatin nephrotoxicity (Nakamura et al., 2010). Furthermore, cell transfected with MATE1 displayed a higher cisplatin uptake than control cells (Nakamura et al., 2010). Interestingly, MATE1 and MATE2-K, another member of MATE family which is solely expressed in human kidneys, seem to transport oxaliplatin with higher affinity than cisplatin (Yokoo et al., 2007), offering a possible explanation of the low oxaliplatin nephrotoxicity. As outlined above, inhibition of OCT2 may be a protective strategy against cisplatin nephrotoxicity. However, some inhibitors of OCT2 such as cimetidine (Matsushima et al., 2009) and ondansetron (Li et al., 2013) interact with higher potency with MATE1, blocking cisplatin efflux from the cells and potentially increasing cisplatin renal toxicity. Indeed, co-treatment of mice with cisplatin and cimetidine was effective in protecting the animals from ototoxicity but not from nephrotoxicity (Ciarimboli et al., 2010).

There are some investigations suggesting a role of novel organic cation transporters (OCTNs) for oxaliplatin transport. These transporters are expressed on the apical membrane of renal proximal tubule cells (Ciarimboli, 2008) and in rat DRGs (Jong et al., 2011). When transfected in human embryonic kidney cells, rat and human OCTN1 and OCTN2 mediate significant oxaliplatin uptake (Jong et al., 2011), suggesting that OCTNs are involved in oxaliplatin neurotoxicity. Apart from these not directly ATP-dependent transporters, multidrug resistance-associated protein 2 (Mrp2) transporter seems to be involved in the efflux of cisplatin and its conjugates from kidney cells, and for this reason to play an important role for control of cisplatin renal toxicity (Wen et al., 2014).

In conclusion, cellular transport of platinum derivatives is mediated by several transport systems. Some transporters, such as OCTs, are specifically expressed in organs, which are damaged by antitumor therapy with platinum derivatives. For this reason, they may be a target for protective intervention. However, an efficient protection can be only reached by specific inhibition of OCTs.

### Conflict of interest statement

The authors declare that the research was conducted in the absence of any commercial or financial relationships that could be construed as a potential conflict of interest.
